# Hierarchical clustering of PI3K and MAPK pathway proteins in breast cancer intrinsic subtypes

**DOI:** 10.1111/apm.13026

**Published:** 2020-02-27

**Authors:** Dinja T. Kruger, Mark Opdam, Joyce Sanders, Vincent van der Noort, Epie Boven, Sabine C. Linn

**Affiliations:** ^1^ Department of Medical Oncology Amsterdam UMC Vrije Universiteit Amsterdam/Cancer Center Amsterdam Amsterdam The Netherlands; ^2^ Division of Molecular Pathology The Netherlands Cancer Institute Amsterdam The Netherlands; ^3^ Department of Pathology The Netherlands Cancer Institute Amsterdam The Netherlands; ^4^ Division of Biometrics The Netherlands Cancer Institute Amsterdam The Netherlands; ^5^ Department of Medical Oncology The Netherlands Cancer Institute Amsterdam The Netherlands; ^6^ Department of Pathology University Medical Centre Utrecht Utrecht University Utrecht The Netherlands

**Keywords:** Breast cancer intrinsic subtypes, phosphatidylinositol‐3‐kinase, mitogen‐activated protein kinase, hierarchical clustering, HER2‐positive breast cancer, triple‐negative breast cancer

## Abstract

The phosphatidylinositol‐3‐kinase (PI3K) and mitogen‐activated protein kinase (MAPK) pathways are frequently activated in breast cancer. We recently demonstrated the importance of analyzing multiple proteins as read‐out for pathway activation in ER+/HER2− breast cancer, since single proteins are known to provide insufficient information. Here, we determined pathway activation in other primary breast cancer intrinsic subtypes derived from postmenopausal patients. Tumor blocks were recollected, and immunohistochemistry was performed using antibodies against PTEN, p‐AKT(Thr308), p‐AKT(Ser473), p‐p70S6K, p‐4EBP1, p‐S6RP(Ser235/236) and p‐ERK1/2, followed by unsupervised hierarchical clustering. In 32 ER+/HER2+, 37 ER−/HER2+ and 74 triple‐negative breast cancer patients, subgroups were identified with preferentially activated (*A*) and preferentially not activated (*N*) proteins. These subgroups likely reflect tumors with differences in biological behavior as well as treatment outcome.

**Abbreviations**
ER+: ER‐positive
HER2: human epidermal growth factor receptor 2
MAPK: mitogen‐activated protein kinase
mTOR: mammalian target of rapamycin
PFS: progression‐free survival
PI3K: phosphatidylinositol‐3‐kinase
PR: progesterone receptor
TMA: tissue microarray
TNBC: triple‐negative breast cancer

The phosphatidylinositol‐3‐kinase (PI3K)/AKT/mammalian target of rapamycin (mTOR) and the mitogen‐activated protein kinase (MAPK) pathways play important roles in breast cancer pathophysiology 1. Activation of and/or alterations in these pathways differ among breast cancer intrinsic subtypes 2, 3. For instance, alterations in PTEN are more common in triple‐negative breast cancer (TNBC) varying between 35% and 67% compared with ER‐positive (ER+) breast tumors (29‐44%) and HER2‐positive (HER2+) breast carcinomas (19–22%) 4. p‐AKT(Ser473) is often overexpressed in luminal‐like breast cancer (78%) and HER2+ breast cancer (80%), while slightly lower percentages are observed in TNBC (58–62%) 5, 6. The MAPK pathway seems more important in the development of TNBC 1. For example, phosphorylation of ERK1/2 was more common in TNBC compared with other breast cancer subtypes 7.

Since PI3K and MAPK pathway activation is increasingly recognized as important in the growth and metastatic potential of breast cancer, a variety of inhibiting drugs have been developed against targets in these pathways 8, 9. The best example so far is everolimus, an mTOR inhibitor registered for the treatment of ER+/HER2− advanced breast cancer 10. Everolimus has also been investigated in HER2+ breast cancer patients. The addition of everolimus to trastuzumab plus paclitaxel in the HR‐negative, HER2+ breast cancer patients in the BOLERO‐1 study showed a clinically relevant progression‐free survival (PFS) prolongation of 7.2 months compared to patients without everolimus 11, although this difference did not cross the protocol‐specified significance threshold of p = 0.0044. In trastuzumab‐resistant, HER2+ advanced breast cancer patients, the addition of everolimus to trastuzumab plus vinorelbine significantly prolonged PFS 12.

PI3K inhibitors are also being investigated in clinical trials for breast cancer subtypes. The PI3K inhibitor alpelisib in combination with fulvestrant has already been approved by the FDA in the US for men and postmenopausal women, with hormone receptor positive, HER2−, PIK3CA‐mutated metastatic breast cancer. This approval was based on the results of the phase 3, randomized, double‐blind, placebo‐controlled SOLAR‐1 study, demonstrating a longer median PFS of 11.0 months of alpelisib plus fulvestrant compared with 5.7 months of placebo plus fulvestrant 13. Results on the PI3K inhibitors buparlisib and pictilisib were less successful. The BELLE‐4 has been discontinued after an interim analysis showed no benefit of the addition of buparlisib to paclitaxel in TNBC 14. In the PEGGY study comparable results for paclitaxel with pictilisib or placebo have been shown in ER+/HER2 − breast cancer 15. In one phase 2 trial with the AKT inhibitor ipatasertib, TNBC patients who received ipatasertib showed PFS benefit compared to those receiving placebo, but further investigation is needed 16.

Feedback loops and cross‐talks between the PI3K and MAPK pathway exist, 1, rendering a single marker at risk to produce false‐positive or false‐negative results when used as read‐out for activation of a pathway. Therefore, the usefulness of single markers for treatment selection is under discussion 17. Previously, our group has demonstrated in ER+/HER2 − breast cancer patients that hierarchical clustering of seven PI3K and/or MAPK proteins has a better potential to discriminate tumors with/without pathway activation than a single marker 17. This method of multiple protein analysis by immunohistochemistry is likely a better read‐out of activated proteins of the PI3K and MAPK pathway and has not yet been explored in other breast cancer intrinsic subtypes.

In the era of molecular drug development for breast cancer it appears that selected patients may benefit from PI3K and/or MAPK pathway inhibitors because of which proper biomarkers are required. Therefore, we carried out unsupervised hierarchical clustering and show for the first time how seven proteins of these pathways cluster in ER+/HER2+, ER−/HER2 + and in TNBC cases. This approach may indicate the presence of tumors with a specific biological behavior and with differences in treatment outcome. Our new method might guide future clinical trials and individualized treatment decisions.

## Materials and Methods

We recollected formalin‐fixed paraffin‐embedded tumor tissue blocks from postmenopausal stage I‐III patients presenting with primary breast cancer who participated in the IKA trial. A detailed description has been published elsewhere 17, 18, 19. This trial was approved by the central ethics committee of the Netherlands Cancer Institute. All patients gave informed consent. Briefly, the IKA trial addressed the putative benefit of adjuvant tamoxifen versus nil. None of the patients received adjuvant chemotherapy or trastuzumab. For immunohistochemistry, we used coded archival pathology left‐over material for which no additional consent was required according to Dutch legislation 20. Tumor tissue was handled according to the Dutch code of conduct for responsible use of human tissue in the context of health research 21. This study complied with reporting recommendations for tumor marker prognostic studies (REMARK) criteria 22.

**Figure 1:**
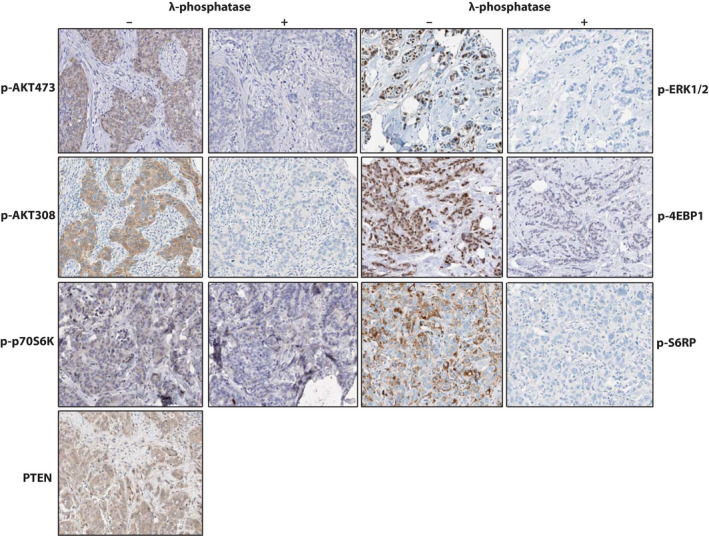
Representative immunohistochemistry images. For PTEN, p‐p70S6K, p‐AKT473, p‐AKT308, p‐4EBP1, p‐ERK1/2 and p‐S16RP, representative immunostaining images are shown. The panels beneath ‘–‘ represent positive TMA cores without previous λ‐phosphatase treatment. The panels beneath ‘+‘ represent positive TMA cores after λ‐phosphatase treatment resulting in negative staining. PTEN was not a phospho‐staining and, therefore, only a positive TMA core was shown.

Tumor material was available from 41 ER+/HER2+, 44 ER−/HER2+ and 98 TNBC patients. Tissue microarrays (TMAs) were constructed using three 0.6 mm cores. TMAs were stained for HER2, ER and progesterone receptor (PR). ER and PR were considered positive if nuclear staining was shown in ≥10% of tumor cells. If membranous staining was DAKO score 3, HER2 was considered positive. In case of DAKO score 2, HER2 amplification had to be confirmed by chromogenic *in situ* hybridization for HER2 positivity. Tumor grade was scored on a hematoxylin‐eosin stained slide according to the modified Bloom–Richardson scoring system 23. Both tumor grade and histological subtype were revised by a pathologist. Staining and scoring for PTEN, p‐AKT(Thr308), p‐AKT(Ser473), p‐p70S6K, p‐4EBP1, p‐S6RP(Ser235/236) and p‐ERK1/2 (all from Cell Signaling Technology, Danvers, MA, US) have been described previously 17, 19, 24, and procedures are summarized in Table S1. Typical IHC staining results, including a control for phosho‐specificity, are shown in Fig. 1. Distribution of expression of each of the seven proteins was investigated for clinicopathological characteristics in all three subtypes by analyzing the median expression of each protein per characteristic.

The hierarchical clustering method has been published before 17. Briefly, patients were selected for which continuous scorings of all seven proteins were available [ER+/HER2+ (n = 32), ER−/HER2+ (n = 37), TNBC (n = 74)]. In these patient subsets, scores were normalized for each specific protein by dividing the IHC scoring result by the standard deviation of the score for that protein. Then, unsupervised hierarchical clustering was carried out. Subgroups were formed within the heatmap cluster reflecting relatively high protein activation (red boxes) and showing less activation (black boxes). Clinicopathological characteristics and recurrence‐free interval (RFI) events, the later defined as an occurrence of a local, regional or distant recurrence or breast cancer‐specific death, were visualized together with the heatmaps. The heatmaps were generated using R for statistics (Windows version 3.3.1).

## Results

**Figure 2:**
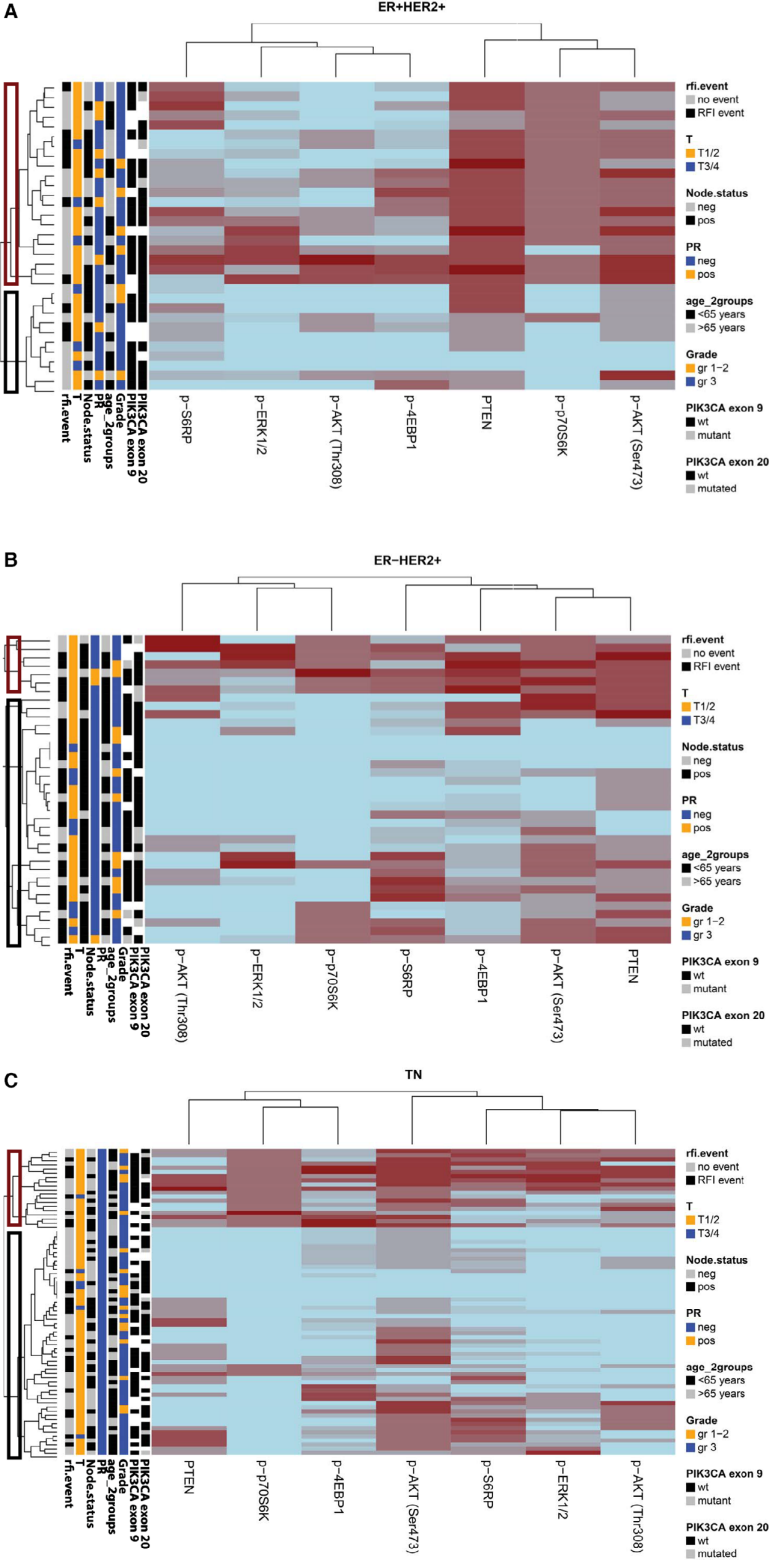
Hierarchical clustering of seven proteins visualized in a heatmap in (A) ER+/HER2 + tumors, (B) ER‐/HER2 + tumors and (C) TN tumors. Red boxes indicate a higher score in activation. Black boxes indicate a lower score and, therefore, less activation of the corresponding protein. Abbreviations: p‐ERK1/2, phosphorylated extracellular signal‐regulated kinase 1 and 2; p‐S6RP, phosphorylated 40S ribosomal protein S6; p‐p70S6K, phosphorylated p70 ribosomal protein S6 kinase, p‐AKT: phosphorylated AKT at phospho‐sites Thr308 and Ser473; p‐4EBP1, initiation factor eukaryotic initiation factor 4E binding protein 1; PTEN, phosphatase and tensin homolog; RFI event, recurrence‐free interval event, gray: no RFI event, black block: RFI event; T: T stage, orange: T stage 1–2, blue: T stage 3–4; Node status: lymph node status, gray: negative, black: positive; PR: progesterone receptor status, orange: positive, blue: negative; Grade, orange: grade 1–2, blue: grade 3; *PIK3CA* exon 9/20: PI3K mutation status, black: wild type, gray: mutated, white: no result available.

Hierarchical clustering analysis of ER+/HER2+, ER−/HER2+ and TNBC cases with expression levels of the seven proteins was followed by the generation of heatmaps in, respectively, 32, 37 and 74 patients (Fig. 2). Clinicopathological characteristics of these patients were generally comparable with the original trial population (Table 1). ER‐ breast cancer, when compared to ER+ disease, contained a higher number of patients aged <65 years. The three breast cancer subtypes had a high incidence of histological grade 3 tumors. HER2+ tumors were more often lymph‐node positive and T3‐4 compared with TNBC.

**Table 1:** Distribution of clinicopathological characteristics of patients presented in the various heatmap subgroups as well as in the original data set of patients with tumor material available and in the total IKA trial population

	ER+/HER2 + population N (%)^a^	ER−/HER2 + population N (%)^b^	TN population N (%)^c^	Patients with tumor material available N (%)	Total study population N (%)
Total	32 (100)	37 (100)	74 (100)	739 (100)	1662 (100)
Age					
<65	15 (47)	24 (65)	47 (64)	378 (51)	869 (52)
≥65	17 (53)	13 (35)	27 (36)	361 (49)	793 (48)
Lymph node status					
Negative	13 (41)	6 (16)	40 (54)	393 (53)	901 (54)
Positive	19 (59)	31 (84)	34 (46)	346 (47)	761 (46)
T stage					
T1–2	26 (81)	29 (78)	68 (92)	659 (89)	1482 (89)
T3–4	6 (19)	8 (22)	6 (8)	80 (11)	180 (11)
Grade					
Grade 1–2	7 (22)	11 (30)	18 (24)	435 (59)	435 (59)^d^
Grade 3	25 (78)	26 (70)	56 (76)	304 (41)	304 (41)^d^
Histological subtype					
Ductal	28 (88)	34 (92)	61 (82)	540 (89)	540 (89)^d^
Lobular	2 (6)	0 (0)	2 (3)	66 (11)	66 (11)^d^
HER2 status					
Negative	0 (0)	0 (0)	74 (100)	594 (88)	594 (88)^d^
Positive	32 (100)	37 (100)	0 (0)	85 (12)	85 (12)^d^
PR status					
Negative	24 (75)	34 (92)	74 (100)	414 (57)	346 (40)^e^
Positive	8 (25)	3 (8)	0 (0)	304 (43)	513 (60)^e^
ER status					
Negative	0 (0)	37 (100)	74 (100)	159 (23)	311 (23)^f^
Positive	32 (100)	0 (0)	0 (0)	563 (77)	1014 (77)^f^

a Subgroup of ER‐positive, HER2‐positive patients used to generate the ER+/HER2+ heatmap

b Subgroup of ER‐negative, HER2‐positive patients used to generate the ER−/HER2+ heatmap.

c Subgroup of triple‐negative (TN) patients used to generate the TN heatmap.

d Only revised scorings from 739 patients of the IKA trial population from whom tumor tissue could be obtained were available.

e Determined by progesterone receptor (PR) ligand binding assay in original trial, missing data of 803 patients.

f Determined by estrogen receptor (ER) ligand binding assay in original trial, missing data of 337 patients.

In all heatmaps, tumor groups with preferably more (red boxes) or less (black boxes) activated PI3K and/or MAPK pathways could be distinguished (Fig. 2). Remarkably, in ER+/HER2+ disease there was a group of tumors with only minor expression of the downstream proteins (Fig. 2A, black box) suggesting that HER2 positivity does not always correlate with an activated downstream PI3K and/or MAPK pathway. A similar finding was noticed in the ER‐/HER2+ group (Fig. 2B). The TNBC patients (Fig. 2C) in the activated subgroup seemed to have a lower T stage and less *PIK3CA* mutations in exon 9 and their tumors showed in particular higher expression of p‐AKT(Thr308), p‐ERK1/2, p‐p70S6K and p‐4EBP1 compared with tumors in the less activated subgroup. An impression of the distribution of RFI events in relation to pathway activation is depicted in Fig. 2. In Tables 2, 3, 4, the relation of all seven proteins with clinicopathological factors is demonstrated. The number of patients in each group is too small to draw statistically robust conclusions on disease outcome.

**Table 2:** Distribution of median scores of PI3K and/or MAPK pathway proteins per clinicopathological characteristic of ER+/HER2 + breast cancer patients

	PTEN	p‐AKT(Thr308)	p‐AKT(Ser473)	p‐p70S6K	p‐4EBP1	p‐S6RP	p‐ERK1/2
	Median	Median	Median	Median	Median	Median	Median
Age							
<65	2	0	1	1	30	30	0
≥65	2	0	2	1	20	20	20
Lymph node status							
Negative	2	1	2	1	30	50	30
Positive	2	0	1	1	20	10	0
T stage							
T1–2	2	0.5	2	1	30	30	20
T3–4	2	0	1.5	0.5	0	5	5
Grade							
Grade 1–2	2	0	2	0	30	20	20
Grade 3	2	0	2	1	20	30	10
Progesterone receptor							
Negative	2	0	1.5	1	20	30	10
Positive	2	0.5	2	1	45	30	15
*PIK3CA mutation *exon 9							
Wild type	2	0	2	1	25	30	10
Mutated	–	–	–	–	–	–	–
*PIK3CA mutation *exon 20							
Wild type	2	0	2	1	25	30	10
Mutated	2	1	2	1	20	30	30

See Table S1 for scoring read‐out.

**Table 3:** Distribution of median scores of PI3K and/or MAPK pathway proteins per clinicopathological characteristic of ER−/HER2 + positive breast cancer patients

	PTEN	p‐AKT(Thr308)	p‐AKT(Ser473)	p‐p70S6K	p‐4EBP1	p‐S6RP	p‐ERK1/2
	Median	Median	Median	Median	Median	Median	Median
Age							
<65	1	0	2	0	20	20	0
≥65	1	0	1	0	10	20	0
Lymph node status							
Negative	1.5	1	1.5	0	55	70	0
Positive	1	0	2	0	10	20	0
T stage							
T1–2	1	0	2	0	30	40	10
T3–4	1	0	1	0	10	5	0
Grade							
Grade 1–2	1	0	1	0	20	20	10
Grade 3	1	0	2	0	20	20	0
Progesterone receptor status							
Negative	1	0	1	0	20	20	0
Positive	2	1	2	1	70	70	10
*PIK3CA mutation* exon 9							
Wild type	1	0	1.5	0	20	20	0
Mutated	2	0	2	1	30	20	10
*PIK3CA mutation* exon 20							
Wild type	1	0	1	0	25	20	0
Mutated	1	1	2	0	10	20	0

**Table 4:** Distribution of median scores of PI3K and/or MAPK pathway proteins per clinicopathological characteristic of triple‐negative breast cancer patients

	PTEN	p‐AKT(Thr308)	p‐AKT(Ser473)	p‐p70S6K	p‐4EBP1	p‐S6RP	p‐ERK1/2
	Median	Median	Median	Median	Median	Median	Median
T stage							
T1–2	1	0	2	0	20	30	5
T3–4	0	0	0.5	0	5	15	0
Age							
<65	1	0	2	0	20	30	0
≥65	0	0	2	0	10	10	0
Lymph node status							
Negative	0	0	2	0	20	25	0
Positive	1	0	1.5	0	15	25	5
Grade							
Grade 1–2	0.5	0	1	0	10	30	0
Grade 3	0.5	0	2	0	20	20	0
Progesterone receptor status							
Negative	0.5	0	2	0	20	25	0
Positive	n.a.	n.a.	n.a.	n.a.	n.a.	n.a.	n.a.
*PIK3CA mutation* exon 9							
Wild type	1	0	2	0	20	30	0
Mutated	0	0	0	0	0	0	0
*PIK3CA mutation* exon 20							
Wild type	0	0	2	0	20	20	0
Mutated	1	0.5	1	0	25	35	20

## Discussion

We here demonstrate that unsupervised hierarchical clustering of seven proteins involved in the PI3K and/or MAPK pathways can distinguish tumors within the ER+/HER2+, ER‐/HER2+ and TNBC subtypes with more or less activated PI3K and MAPK pathways.

In the present study, the majority of breast tumors were characterized by grade 3 disease with percentages up to 78%. These percentages are substantially higher than the average of 30–50% grade 3 tumors found in the general breast cancer population. However, the general breast cancer population consists of 70–75% of the prognostic favorable ER+/HER2− subtype 25. In the current study, we analyzed the more aggressive subtypes represented by HER2 positivity or negativity for ER, PR and HER2. Our results are in line with other groups who have published similar grade 3 percentages in comparable patients 25, 26, 27, 28, 29, 30.

Within the three breast cancer intrinsic subtypes, we show by the heatmap analysis that subgroups of tumors exist with pathway activation and with less/no activation. In a previous study, Horii et al 31 have used the clustering method to examine 337 breast cancer patients from various breast cancer intrinsic subtypes with IHC staining scores for p‐AKT(Ser473), cyclin D1, P27, p‐p70S6K, p‐4EBP1 and p‐ERK1/2. They compared subgroups formed by clustering with prognostic factors and found significant relationships with histological subtype, hormone receptor and HER2 status. Since they combined all breast cancer subtypes to generate one heatmap, comparison with our heatmaps is not possible.

HER2 is able to activate both PI3K and MAPK pathways as upstream receptor tyrosine kinase upon dimerization with ErbB family receptor members 32 and, therefore, our result on the presence of HER2‐positive tumor samples with relatively less to no activation of the PI3K and/or MAPK pathways was unexpected. Whether the activation status, more specifically PTEN protein expression, is predictive of response to anti‐HER2‐based therapies is subject of current research. The group of Jensen 33 have reported that patients with a tumor either with PTEN low or with *PI3K* mutations had a significantly worse survival despite adequate adjuvant chemotherapy and trastuzumab. Stern et al. 34 have also found that complete absence of PTEN staining in tumor cells was associated with a significant decrease in disease‐free survival and overall survival, but that these patients may still derive benefit from trastuzumab. In contrast, Perez et al. 35 have described that neither disease‐free survival nor adjuvant trastuzumab benefit was related to PTEN protein status. Loibl et al. 36 have reported a significantly higher pathological complete remission rate in HER2+, PTEN‐high tumors upon neo‐adjuvant trastuzumab‐containing chemotherapy. Of note, only one third of patients receiving adjuvant trastuzumab derive overall survival benefit 37. One factor of influence on prognosis in HER2+ breast cancer is the presence of ER or absence of ER 38. Furthermore, it would be of interest to analyze whether trastuzumab‐based treatment outcome in patients with HER2+ tumors differs between subgroups with an activated and not activated PI3K and/or MAPK pathway. This would shed more light on whether inhibition of downstream growth factor signaling pathways is a relevant working mechanism of trastuzumab, next to antibody‐dependent cell‐mediated cytotoxicity 39.

Activated and less activated subgroups based on PI3K and/or MAPK protein expression profiles could be distinguished in our postmenopausal TNBC patient cohort. TNBC is a heterogeneous disease and many tumors contain deregulated PI3K and/or MAPK pathways 1, 4, 5, 7. It appears that the presence of PI3K mutations in early‐stage TNBC 40 as well as positive expression of p‐AKT(Ser473) or p‐ERK1/2 in node‐positive TNBC are potentially favorable prognostic factors 41. Current research is dedicated toward the development of drugs directed against these pathways to improve dismal prognosis in TNBC patients 42, 43. These therapies might specifically be effective in tumors assigned to the more activated subgroup.

Concluding, hierarchical clustering of individual breast cancer intrinsic subtypes can distinguish tumors with more or less activated PI3K and/or MAPK pathways. Whether this approach is of value for an individualized treatment choice deserves further exploration in well‐defined cohorts of primary breast cancer patients 44.

We would like to acknowledge the Core Facility Molecular Pathology & Biobanking (CFMPB) of the Netherlands Cancer Institute for supplying tissue material and/or laboratory support. We would like to acknowledge Bram Thijssen, bioinformatician at the Molecular Carcinogenesis Division of the Netherlands Cancer Institute, for independently reproducing the hierarchical clustering analysis.

## Funding

This work was supported by grants from TI Pharma [project number T3‐502] and from A Sister’s Hope.

## Conflict Of Interest

SCL is an advisory board member for AstraZeneca, Cergentis, Novartis, Roche and Sanofi. SCL received institutional research support funding from Adienne, Amgen, AstraZeneca, Genentech, Roche, Tesaro and Sanofi.

## Authors' Contributions

DTK, SCL and EB were involved in the concept and design of the study. MO and JS contributed to data collection and generation. DK, VN, EB and SCL contributed to the analysis and interpretation of the data. DK, with supervision from EB and SCL, drafted the manuscript. All authors revised the manuscript and approved the final version.

## Availability Of Data And Material

The data that support the findings of this study are available from the corresponding author upon reasonable request.

## Supporting information


**Table S1.** Antibodies used for immunohistochemistry, scoring procedures and kappa coefficients to determine the interobserver variability.
