# Supplementary material for: Hierarchical clustering of PI3K and MAPK pathway proteins in breast cancer intrinsic subtypes
Source: APMIS. 2020 Feb 27;128(4):298–307. doi: 10.1111/apm.13026 (PMC7317370; doi:10.1111/apm.13026)
Supplement: Supplementary file 1 — Table S1. Antibodies used for immunohistochemistry, scoring procedures and kappa coefficients to determine the interobserver variability. [file APM-128-298-s001.docx]

## Supplemental Material

## Hierarchical clustering of PI3K and MAPK pathway proteins in breast cancer intrinsic subtypes

Dinja T Kruger, Mark Opdam, Joyce Sanders, Vincent van der Noort, Epie Boven, Sabine C Linn

**Table S1**. Antibodies used for immunohistochemistry, scoring procedures and kappa coefficients to determine the interobserver variability

| **Antibody** | **Art. No. (clone)^1^** | **Scoring system** | **Scoring for hierarchical clustering** | **Comparable cores from N patients for kappa calculation** | **Cut-off for binary score for kappa calculation** | **Kappa for binary score** |
| --- | --- | --- | --- | --- | --- | --- |
| PTEN | 9559 (138G6) | cytoplasmic intensity | 0 – 3 | 126 | 0 *vs* 1-3 | 0.75 |
| p-AKT (Thr308) | 2965 (C31E5E) | cytoplasmic intensity | 0 – 3 | 133 | 0 *vs* 1-3 | 0.53 |
| p-AKT (Ser473) | 4060 (D9E) | cytoplasmic intensity | 0 – 3 | 94 | 0-1 *vs* 2-3 | 0.70 |
| p-p70S6K (Thr389) | 9206 (1A5) | cytoplasmic intensity | 0 – 3 | 97 | 0 *vs* 1-3 | 0.44 |
| p-4EBP1 (Ser65) | 9456 (174A9) | percentage of tumour cells with nuclear staining | 0 – 100% | 112 | 0-50 *vs* 51-100 | 0.59 |
| p-S6RP (Ser235/236) | 2211 | percentage of tumour cells with cytoplasmic and membranous staining | 0 – 100% | 85 | 0-19 *vs* 20-100 | 0.55 |
| p-ERK1/2 (Thr202/Tyr204) | 4370 (D13.14.4E) | proportion of tumour cells with nuclear staining | 0 – 3 | 69 | negative *vs* positive | 0.85 |

^1^all from Cell Signaling Technology, Danvers, MA, US
